# One Gene and Two Proteins: a Leaderless mRNA Supports the Translation of a Shorter Form of the *Shigella* VirF Regulator

**DOI:** 10.1128/mBio.01860-16

**Published:** 2016-11-08

**Authors:** Maria Letizia Di Martino, Cédric Romilly, E. Gerhart H. Wagner, Bianca Colonna, Gianni Prosseda

**Affiliations:** a Istituto Pasteur Italia-Fondazione Cenci Bolognetti, Department of Biology and Biotechnology C. Darwin, Sapienza Università di Roma, Rome, Italy; b Department of Cell and Molecular Biology, Biomedical Center, Uppsala University, Uppsala, Sweden

## Abstract

VirF, an AraC-like activator, is required to trigger a regulatory cascade that initiates the invasive program of *Shigella* spp., the etiological agents of bacillary dysentery in humans. VirF expression is activated upon entry into the host and depends on many environmental signals. Here, we show that the *virF* mRNA is translated into two proteins, the major form, VirF_30_ (30 kDa), and the shorter VirF_21_ (21 kDa), lacking the N-terminal segment. By site-specific mutagenesis and toeprint analysis, we identified the translation start sites of VirF_30_ and VirF_21_ and showed that the two different forms of VirF arise from differential translation. Interestingly, *in vitro* and *in vivo* translation experiments showed that VirF_21_ is also translated from a leaderless mRNA (llmRNA) whose 5′ end is at position +309/+310, only 1 or 2 nucleotides upstream of the ATG84 start codon of VirF_21_. The llmRNA is transcribed from a gene-internal promoter, which we identified here. Functional analysis revealed that while VirF_30_ is responsible for activation of the virulence system, VirF_21_ negatively autoregulates *virF* expression itself. Since VirF_21_ modulates the intracellular VirF levels, this suggests that transcription of the llmRNA might occur when the onset of the virulence program is not required. We speculate that environmental cues, like stress conditions, may promote changes in *virF* mRNA transcription and preferential translation of llmRNA.

## IMPORTANCE

*Shigella* spp. are a major cause of dysentery in humans. In bacteria of this genus, the activation of the invasive program involves a multitude of signals that act on all layers of the gene regulatory hierarchy. By controlling the essential genes for host cell invasion, VirF is the key regulator of the switch from the noninvasive to the invasive phenotype. Here, we show that the *Shigella virF* gene encodes two proteins of different sizes, VirF_30_ and VirF_21_, that are functionally distinct. The major form, VirF_30_, activates the genes necessary for virulence, whereas the minor VirF_21_, which shares the C-terminal two-thirds of VirF_30_, negatively autoregulates *virF* expression itself. VirF_21_ is transcribed from a newly identified gene-internal promoter and, moreover, is translated from an unusual leaderless mRNA. The identification of a new player in regulation adds complexity to the regulation of the *Shigella* invasive process and may help development of new therapies for shigellosis.

## INTRODUCTION

*Shigella* spp. are highly adapted human pathogens that cause bacillary dysentery (1). The sophisticated infectious strategy of *Shigella* depends on the capacity to invade, disrupt, and cause inflammatory destruction of the intestinal epithelial barrier (2, 3). Activation of the invasive program is exceptionally complex and involves many signals affecting gene regulation at different levels. A key factor is VirF, an AraC-like transcription factor (TF) whose expression is activated as *Shigella* bacteria sense entry into the host environment (4, 5). In a cascade model, VirF triggers activation of the *virB* and *icsA* genes. IcsA affects bacterial intracellular spreading, and VirB promotes expression of several virulence genes, including those encoding a type III secretion system (T3SS), its effectors, and the last regulator of the cascade, MxiE (6, 7). Interestingly, MxiE, another AraC-like TF, appears to rely on high-level transcriptional slippage to generate its reading frame from two separate open reading frames (ORFs) (8). The genes *virF*, *icsA*, *virB*, and those controlled by VirB are located on a virulence plasmid (pINV) and are silenced outside the human host (9). At low temperatures, the nucleoid-associated protein H-NS represses transcription of the virulence genes (5). In a temperature-dependent manner, H-NS interacts with two sites within the *virF* promoter, spaced by an intrinsically curved DNA region, to prevent access of RNA polymerase (5, 10, 11). At a permissive temperature (37°C), reduced DNA curvature counteracts H-NS binding (4, 12) and unmasks a binding site for the nucleoid protein FIS to activate *virF* transcription (13). VirF then relieves H-NS-mediated repression of *virB* and *icsA* and directly stimulates transcription (14, 15). By binding upstream of the *virF* promoter between two H-NS sites, VirB also counteracts H-NS-dependent repression of *virF* transcription (16). Expression of *virF* is further modulated by integration host factor (IHF) (17) and other environmental factors, such as pH and osmolarity (7), and is affected by tRNA modifications (18).

The relevance of *virF* activation for the invasive program is further supported by posttranscriptional regulation of *icsA.* RnaG is a *cis*-encoded antisense RNA that promotes premature termination of the *icsA* mRNA (19). VirF binds the RnaG promoter and decreases *rnaG* expression (14). Thus, VirF plays a dual role: (i) it relieves H-NS-mediated repression to activate *icsA* transcription, and (ii) it represses RnaG transcription, thus increasing the level of *icsA* mRNA (14). VirF also globally activates the expression of chromosomal genes in both *Shigella* and *Escherichia coli*. In particular, VirF appears to play a role in shaping the *Shigella* transcriptional program to better match the requirements of an effective intracellular life (20–22).

Like other members of the AraC family of transcriptional regulators, VirF has a conserved, carboxy-terminal DNA-binding domain with two helix-turn-helix (HTH) motifs. AraC-like proteins are typically insoluble and, accordingly, problems with VirF purification have hampered biochemical studies (23). Mutagenesis experiments indicated that the N-terminal domain of VirF promotes dimerization while C-terminal HTH2 motif mutants are nonfunctional (24).

While attempting a thorough characterization of VirF, we found that the *virF* mRNA (R1) is subject to differential translation, giving rise to two forms of VirF. VirF_30_ activates the virulence system and some chromosomal genes, whereas VirF_21_ exerts negative feedback control on *virF* expression itself.

Moreover, we identified a second *virF* mRNA species (R2) with a 5′ end at position nucleotide (nt) +309/+310. This leaderless yet translation-competent mRNA is transcribed from a gene-internal promoter. Possible implications in an interplay between environmental sensing and virulence gene expression are discussed.

## RESULTS

### The *virF* gene encodes two independently translated proteins, VirF_30_ and VirF_21_.

**FIG 1:**
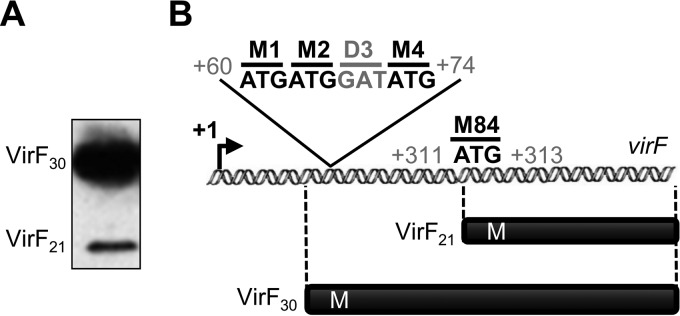
Detection of two VirF protein variants. (A) Western blot analysis with anti-FLAG antibody of whole-cell extracts of *S. flexneri* M90T carrying *virF*-3×FLAG. Two forms are indicated, VirF_30_ and VirF_21_. (B) Schematic organization of the *virF* gene of *Shigella*. The methionine (M) codons relevant for this study are highlighted. The transcription start site (+1) was identified previously (5).

Earlier experiments on *E. coli* minicells carrying the *virF* gene on recombinant plasmids from *Shigella flexneri* and *Shigella sonnei* indicated two main VirF protein forms of about 30 and 21 kDa and a minor form of 27 kDa (25, 26). The significance of the 27- and 21-kDa forms remained unclear, and it seemed possible that they were degradation products of full-length VirF (27). To analyze which VirF forms are present in *Shigella*, a 3×FLAG tag sequence was inserted at the 3′ end of the *S. flexneri* M90T *virF* ORF. Western blot analysis (Fig. 1A) confirmed that two VirF proteins, VirF_30_ (30 kDa) and VirF_21_ (21 kDa), are expressed by *S. flexneri*. The 27-kDa form was not detected.

**FIG 2:**
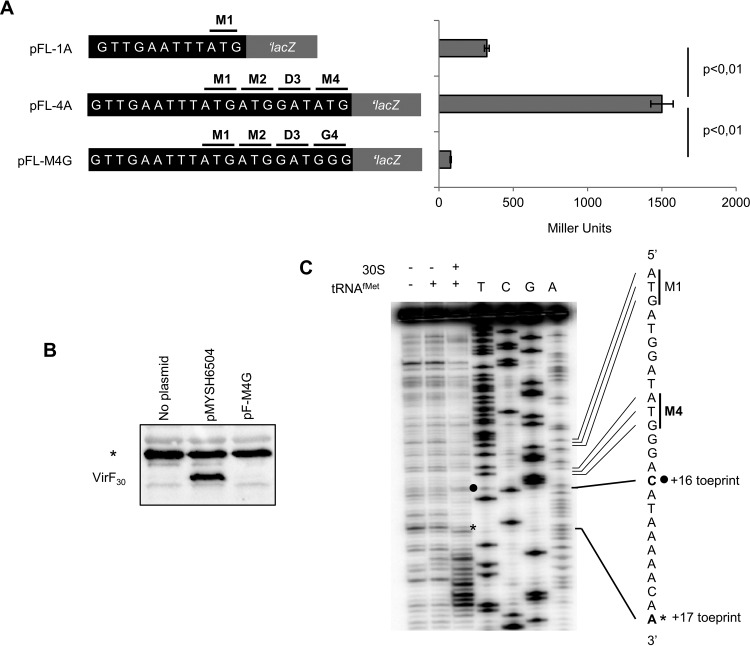
Identification of the translation start codon of VirF_30_. (A, left) Schematic of the pFL plasmids used. Plasmids pFL-1A and pFL-4A carry a translational fusion between the 5′-UTR of *virF* mRNA after Met1 (pFL-1A) or Met4 (pFL-4A), in frame with *lacZ*. (Right) pFL-M4G, the Met4 codon, was replaced by Gly (ATG to GGG). β-Galactosidase activities of *E. coli* strain DH10b carrying the same *virF-lacZ* plasmids are shown. Strains harboring the pRS414 vector showed very low β-galactosidase activity levels (2 to 4 Miller units) relative to the values obtained. Values are averages of three experiments, and standard deviations are indicated. (B) Western blot with VirF antibodies on extracts from MG1655 harboring pMYSH6504, a plasmid carrying the wt *virF* gene of *S. flexneri*, or pF-M4G (pMYSH6504 with the M4G substitution). The asterisk indicates unspecific cross-hybridization with a protein in the extract. (C) Toeprint assay results on the +1 *virF* mRNA (see Materials and Methods). The mRNA was incubated alone (lane 1), with 30S (lane 2), and with 30S and tRNA^fMet^ (lane 3). Toeprints at position +16 from ATG1 and at +17 from ATG4 are indicated by a black circle and asterisk, respectively. Sequencing ladders were generated with the same 5′-end-labeled primer.

The sequence of the *virF* gene contains three putative start codons, all in the same frame, for VirF_30_ and an internal ATG codon, consistent with independent translation of VirF_21_ (25). Thus, we determined at which ATG codons VirF_30_ and VirF_21_ translation initiates. In the absence of a recognizable Shine-Dalgarno (SD) sequence upstream, prediction of the ATG encoding the N-terminal methionine of VirF_30_ was difficult. Thus, each of the ATG codons (codons 1, 2, and 4; codon 3 encodes Asp) (Fig. 1B) was tested for translation initiation activity by using plasmids carrying the *virF* promoter followed by a *virF*-*lacZ* translational fusion. Plasmid pFL-4A is fused in frame after the fourth *virF* codon (third Met codon), and pFL-1A is fused after the first ATG (Fig. 2A). β-Galactosidase activities indicated that the construct with all three ATGs has ≈5-fold-higher activity than the one fused after ATG_1_. Thus, ATG_2_ and/or ATG_4_ appear to be required for high translation of VirF_30_, and ATG_1_ gives a minor contribution. ATG_4_, which has a short upstream SD-like (GAA) sequence, was tested by introducing an ATG_4_ → GGG (Gly) mutation into pFL-4A. This plasmid, pFL-M4G, in which ATG_1_ and ATG_2_ are still present, gave very low reporter gene activity (Fig. 2A), suggesting ATG_4_ as the main VirF_30_ start codon. Western blot analysis supported this. VirF_30_ was produced only from the wild-type (wt) *virF* gene, but not when ATG_4_ had mutated (Fig. 2B, cf. pMYSH6504 and pF-M4G). To corroborate this finding *in vitro*, we used a toeprinting assay to analyze the formation of ribosomal initiation complexes on *virF* mRNA (28). A predominant toeprint was seen 17 nt downstream of AUG_4_ and a minor one 16 nt downstream of AUG_1_ (Fig. 2C), in line with our *in vivo* results (Fig. 2A and B). Additional bands downstream of position +17 of AUG_4_ implicated possible 30S binding-driven structure changes resulting in reverse transcription pauses. In conclusion, translation of VirF_30_ initiates predominantly at ATG_4_. Throughout the remainder of this paper, codon positions are accordingly renumbered, with ATG_4_ as codon 1.

**FIG 3:**
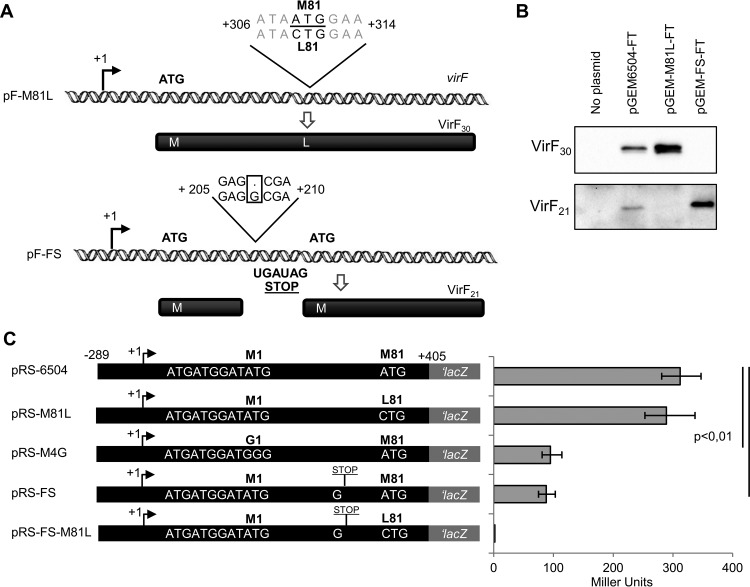
Differential translation of VirF_30_ and VirF_21_. (A) Schematic representation of the constructs used to exclusively produce VirF_30_ or VirF_21_. Sequences relevant for the construction of plasmid pF-M81L (M81L substitution) or plasmid pF-FS (insertion of G at position +208) are highlighted. Plasmids are derivatives of pMYSH6504. (B) Western blot analysis (with anti-FLAG antibody) of extracts of *E. coli* DH10b carrying pGEM-6504-FT (VirF_30_ and VirF_21_), pGEM-M81L-FT (only VirF_30_), or pGEM-FS-FT (only VirF_21_). (C) β-Galactosidase activity levels of the *virF-lacZ* plasmids obtained by fusing a fragment (−289 to +405) of the *virF* gene of pMYSH6504 (pRS-6504), pF-M81L (pRS-M81L), pF-M4G (pRS-M4G), pF-FS (pRS-FS), or pF-FS-M81L (pRS-FS-M81L) as the control, to the promoterless *lacZ* gene of pRS414. Values are averages of three experiments, and standard deviations are indicated.

While searching for a VirF_21_ translation start site, we noticed an in-frame ATG codon within *virF* at position 311 to 313 (relative to +1 of *virF*) (Fig. 1B), consistent with translation of the minor form of VirF. To validate ATG_81_ (formerly ATG_84_) as the start codon for VirF_21_, two mutations were introduced into *virF*, generating a codon change and a frameshift, respectively. To mutate ATG_81_ to a different codon that would retain VirF_30_ function, we changed the ATG (mRNA position 311 to 313) to CTG (Met to Leu; pF-M81L) (Fig. 3A) or to ATC (Met to Ile; pF-M81I). Neither mutation should affect VirF_30_ translation but should abolish independent translation of VirF_21_. Both mutant VirF_30_ proteins were tested for activated expression of *virB* in a *virF*-defective *S. flexneri* strain (M90TFd) (see Table S1 in the supplemental material) carrying plasmids expressing wt VirF, VirF_M81L_, or VirF_M81I_. VirF_M81L_ but not VirF_M81I_ activated *virB* to a level comparable to wt (see Fig. S1 in the supplemental material). Thus, the substitution in VirF_M81I_ impairs VirF_30_ functionality, and therefore only pF-M81L was used in subsequent experiments. Moreover, the exclusive expression of VirF_30_ upon Met → Leu substitution (Fig. 3B) identified ATG_81_ as the start codon for VirF_21_.

To uncouple the translation of VirF_30_ and VirF_21_, we inserted a single G between positions +207 and +208 of *virF* to create a frameshift (pF-FS) into two stop codons (UGA and UAG, +243 to +249), upstream of ATG_81_ of VirF_21_ (Fig. 3A). The wt *virF* gene and its M81L (substitution) and frameshift mutant variants were FLAG tagged, resulting in plasmids pGEM-6504-FT, pGEM-M81L-FT, and pGEM-FS-FT. Western blot analysis of total protein from *E. coli* cells showed that only VirF_30_ is translated from pGEM-M81L-FT and only VirF_21_ is translated from pGEM-FS-FT (Fig. 3B). Western blotting assays with cells with untagged plasmids confirmed this result (see Fig. S2 in the supplemental material). Since premature termination of frameshifted *virF* only abolished the synthesis of VirF_30_ and not that of VirF_21_, both proteins are independently translated.

The relative expression of the two VirF forms was further analyzed by translational *lacZ* fusions. Four *virF-lacZ* fusions enabled us to monitor translation of VirF_21_ (pRS-M4G and pRS-FS) and VirF_30_ (pRS-M81L), in comparison to total wt *virF* mRNA translation (pRS-6504). The β-galactosidase levels from pRS-M4G and pRS-FS were about 3-fold lower than those from pRS-6504 and pRS-M81L, which is congruent with the Western blot results shown in Fig. 3B; under these experimental conditions, VirF_21_ is a minor fraction of the total VirF protein pool.

### VirF_21_ negatively autoregulates the *virF* gene.

What is the function of the independently translated VirF_21_? To test whether VirF_21_ can activate virulence genes, the promoters of *virB* and *icsA* were transcriptionally fused to *lacZ* and transferred to the chromosome of the *E. coli* K-12 strain P90C, generating P90CλB and P90CλA, respectively (see Materials and Methods). Activation by wt VirF_30_ and VirF_21_ (pMYSH6504), VirF_30_ (pF-M81L), and VirF_21_ (pF-FS) was monitored in strain P90C λB or P90C λA.

**FIG 4:**
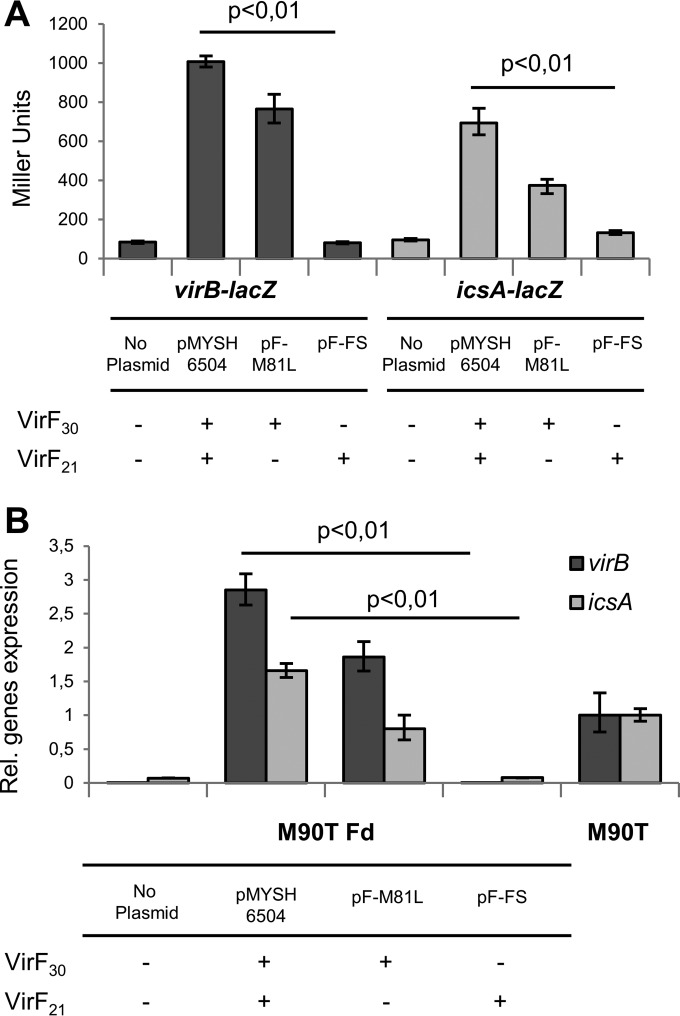
Functional analysis of VirF_30_ and VirF_21_. (A) β-Galactosidase activity of *E. coli* P90C carrying *virB-lacZ* and *icsA-lacZ* transcriptional fusions, transformed with a plasmid expressing VirF_30_ and VirF_21_ (pMYSH6504), only VirF_30_ (pF-M81L), or only VirF_21_ (pF-FS). Values are averages of three experiments, with standard deviations indicated. (B) *In vivo* levels of *virB* and *icsA* mRNA as a function of the two VirF forms, monitored by qRT-PCR in a Δ*virF S. flexneri* strain (M90T Fd) transformed with pMYSH6504 (VirF_30_ and VirF_21_), pF-M81L (VirF_30_), or pF-FS (VirF_21_). Expression levels were monitored in M90T or M90T Fd as controls. Samples were run in triplicate, and error bars show the calculated maximum (RQMax) and minimum (RQMin) levels that represent the standard error of the mean expression level (RQ value).

Figure 4A shows that VirF_30_ alone (pF-M81L) induced the expression of both *lacZ* fusions to a level similar to that in the presence of both VirF_30_ and VirF_21_ (pMYSH6504). VirF_21_ alone (pF-FS) failed to activate (Fig. 4A). Quantitative reverse transcription-PCR (qRT-PCR) results with the *S. flexneri* strain M90T Fd (*virF* defective) carrying the same three plasmids supported this conclusion (Fig. 4B). Thus, a role for VirF_21_ in the activation of the virulence cascade of *Shigella* is ruled out. A qRT-PCR experiment also confirmed that the previously shown VirF-dependent activation of some chromosomal heat shock genes (20) cannot be carried out by VirF_21_ (see Fig. S3 in the supplemental material).

**FIG 5:**
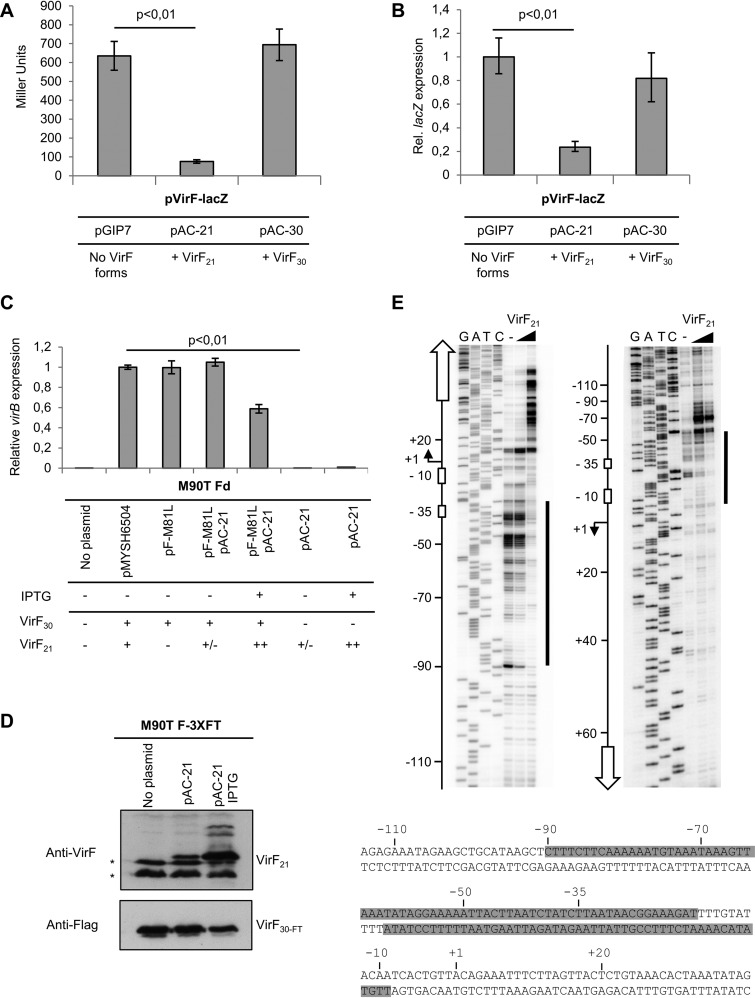
VirF_21_ autoregulates *virF* expression. (A) β-Galactosidase activity of *virF-lacZ* fusions in response to increased levels of VirF_21_ or VirF_30_. Ectopic expression of VirF_21_ or VirF_30_ was obtained in *E. coli* p*_virF_*-*lacZ* strains carrying pAC-21 or pAC-30, respectively. pGIP7, empty vector. Values are averages of three experiments, and standard deviations indicated. (B) *In vivo* levels of *lacZ* mRNA were monitored in the same samples used in the β-galactosidase assay summarized in panel A. Triplicate samples were evaluated, and error bars indicate standard errors of the mean expression levels (RQ values). (C) *In vivo* levels of *virB* mRNA were monitored in the Δ*virF S. flexneri* strain (M90T Fd) carrying pF-M81L (VirF_30_) or ectopically expressing VirF_21_ under IPTG control (pAC-21). Triplicate samples were evaluated; error bars show standard errors of the mean expression levels (RQ values). (D) Western blot analysis of cell extracts of M90T F3xFT carrying pAC-21, with or without ectopic induction of VirF_21_. The level of expression of VirF_30_ was monitored with an anti-FLAG antibody. VirF_21_ induction was monitored with an anti-VirF antibody. Asterisks indicate unspecific cross-hybridization with an unknown protein in the extract. (E) Identification of the VirF_21_ binding site on the *virF* promoter based on DNase I footprinting results. Plasmid pMYSH6504 DNA (41) was incubated with 0, 10 or 20 µl of *in vitro*-translated VirF_21_. The samples were DNase I treated and subsequently analyzed as described in Materials and Methods, using ML-U30 and ML-U29 as primers. Sequencing ladders were generated with the same 5′-end-labeled plus- or minus-strand-specific primers. The VirF_21_-protected site is indicated by vertical black lines and shown by shading on both strands of the *virF* promoter sequence.

To address putative functions of VirF_21_, we investigated its role in positive or negative autoregulation of the *virF* gene. An *E. coli* K-12 strain harboring a P*_virF_*-lacZ fusion (DH10b p*virF*-*lacZ*) was transformed with plasmids that expressed either Ptac promoter-driven VirF_30_ (pAC-30) or VirF_21_ (pAC-21). Figure 5A clearly shows that VirF_21_, but not VirF_30_, strongly repressed *virF* expression, and qRT-PCR on the same samples showed corresponding decreases in *lacZ* mRNA levels in the presence of VirF_21_ (Fig. 5B). To validate VirF_21_-mediated repression of *virF* transcription in *Shigella*, we asked whether increased VirF_21_ levels would reduce the expression of the VirF-activated *virB* gene. qRT-PCR experiments in the *virF*-defective strain M90T Fd expressing VirF_30_ from pF-M81L confirmed severely reduced *virB* transcription upon induction (isopropyl-β-d-thiogalactopyranoside [IPTG]) of VirF_21_ (pAC-21) (Fig. 5C). To monitor the VirF_21_ induction-dependent effect on the VirF protein level, we introduced pAC-21 in the *S. flexneri* strain that contained the 3×FLAG *virF* gene (M90T F3xFT; see above). This setup permitted us to assess the levels of VirF_21_ and VirF_30_ encoded by pINV by FLAG-tagged antibodies as a function of increasing levels of untagged VirF_21_ expressed from pAC-21 (monitored via a halon anti-VirF antibody). Figure 5D shows that increasing the VirF_21_ concentration resulted in a decrease in VirF_30_, confirming that VirF_21_ negatively autoregulates *virF* expression.

In addition, we performed DNase I footprinting by *in vitro*-translated VirF_21_ on both strands of the *virF* promoter region. VirF_21_ was translated in an *in vitro* system (PureSystem) (see Materials and Methods), using a PCR-generated DNA template for *virF_21_*-only transcription and translation. VirF_21_ translation was verified by Western blotting (see Fig. S4 in the supplemental material). Figure 5E indicates that VirF_21_ binding conferred protection of the *virF* promoter region between positions −90 and −20 on the plus strand and approximate positions −60 to −10 on the minus strand and enhanced minus-strand cleavage from about positions −70 to −90. This result, together with data from *in vivo* experiments (Fig. 5A and B), strongly suggests that the transcriptional repression of *virF* by VirF_21_ depends on its direct binding to the consensus *virF* promoter elements.

### Identification of a VirF_21_-encoding leaderless mRNA.

**FIG 6:**
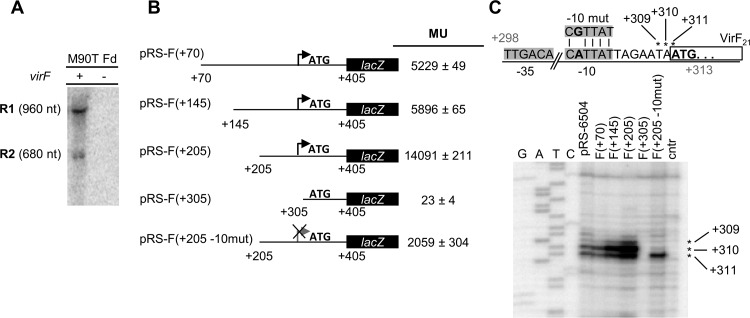
*In vivo* analysis of *virF* transcripts. (A) Northern hybridization of 10 µg of total RNA from *S. flexneri* strain M90TFd, with or without pmysh6504 (*virF* wt) and a *virF*-specific ^32^P-labeled probe indicated two major mRNA variants. (B) Schematic representation of *virF-lacZ* transcriptional fusions carrying truncations of the region upstream of the translational start site of VirF_21_. The ATG for VirF_21_ is indicated as a reference, and the putative promoter is depicted by an arrow. The β-galactosidase activities of the *virF-lacZ* fusion strains were determined. Values reported are in Miller units and represent the averages ± standard deviations of five independent experiments. (C) Schematic representation of the new *virF* promoter. The positions of the −35 and −10 elements are indicated, and the mutated −10 box (−10mut) is shown above. PE analysis results are shown for total RNA extracted from *E. coli* cells carrying the indicated plasmids. Three 5′ ends at position +309, +310, and +311 are indicated by asterisks. The −10 mutation shows only a 5′ end at +311.

The above results showed that two VirF proteins are independently translated. Whether both are translated from the same mRNA, or different versions of *virF* transcripts, was unknown. The possibility of different mRNAs was suggested by two *virF* mRNA variants detected in a Northern blot assay performed with total RNA from strain M90T Fd complemented with the *virF*-encoding pMYSH6504 and with plasmid-free M90T Fd (Fig. 6A). An ≈960-nt band (full-length *virF* mRNA; R1) and an ≈680-nt mRNA that might support translation of VirF_21_ (R2) were visible. To test whether R2 *virF* mRNA is transcribed from a *virF* internal promoter or generated by processing, *virF-lacZ* transcriptional fusions and primer extension (PE) analyses were used. We constructed four *virF-lacZ* fusions starting at positions +70, +145, +205, and +305; all were fused at +405. The β-galactosidase activities clearly indicated the presence of a promoter between +205 and +305; truncation up to position +305 produced background values (Fig. 6B). A promoter was indeed predicted by PromoterHunt (29), with consensus −10 (CATTAT; +298 to +303) and −35 elements (TTGACA; +276 to +289) (Fig. 6C). After mutagenesis of the −10 box [CATTAT to CGTTAT; pRS-F(+205 −10mut)], we observed a severe reduction (≈7-fold) in the β-galactosidase level. This new promoter was further delineated by PE analysis on RNA extracted from *E. coli* cells harboring the different *virF-lacZ* plasmids. This showed 5′ ends at positions +309, +310 (major band), and +311. All three bands were absent in the PE on pRS-F(+305), while with pRS-F(+205 −10mut) the +309/310 bands were not detected. The weaker band at +311 is consistent with a shifted −10 box (data not shown). Thus, the R2 *virF* mRNA is transcribed from a second *virF* promoter, with a transcription start site at position +309/+310.

**FIG 7:**
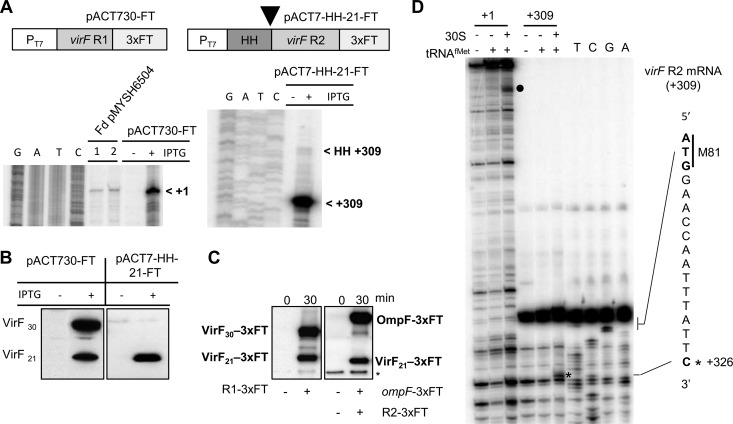
Analysis of VirF_21_ translation from the *virF* R2 transcript. (A) Primer extension analysis of total RNA from BL21(DE3) cells carrying pAC-T730FT or pACT7-HH-21-FT with or without induction using IPTG. The arrowhead indicates the position of hammerhead cleavage. (B) Western blot analysis of total protein extracts from strain BL21(DE3) cells carrying pAC-T730FT or pACT7-HH-21-FT, with or without induction by IPTG. Shown is translation of both VirF_30_ and VirF_21_ in the presence of pACT7-HH-21-FT or of only VirF_21_ in the presence of pACT7-HH-21-FT. (C) *In vitro* translation of *virF* R1-3XFT (start, + 1) and R2-3XFT (start, + 309) mRNAs. Both VirF_30_ and VirF_21_ were translated from *virF* R1-3XFT, but only VirF_21_ was translated from *virF* R2-3XFT mRNA. Asterisk, unspecific cross-hybridization with a protein in the extract. In the blot on the right, we included *ompF* mRNA as an internal canonical, SD-dependent translation control. (D) Toeprint assay results with +1 (full-length) and +309 (leaderless) *virF* mRNAs. The mRNAs were incubated alone (control; lanes 1 and 4), with 30S (lanes 2 and 5), or with 30S and tRNA^−fMet^ (lanes 3 and 6). A specific toeprint was observed on full-length mRNA (+1) near the 5′ end (black circle) (compare with Fig. 2C). A second toeprint, specific to the llmRNA, is at position +326 (black asterisk). Sequencing ladders were generated with the same 5′-end-labeled primer.

The 5′ ends at +309 to 311 and the start codon at +311 to 313 imply that the R2 mRNA is leaderless (Fig. 6C). To test whether the llmRNA is VirF_21_ translation competent, we cloned the sequence corresponding to R2 mRNA, and also the entire R1 mRNA as a control, downstream of a T7 promoter. To ensure a correct 5′ end of the R2 mRNA *in vivo* (5′ U_+309_ as +1) (Fig. 6C), a hammerhead ribozyme sequence downstream of the T7 promoter (see Fig. S5 in the supplemental material) was introduced to generate an R2 mRNA starting at position +309. The plasmids carrying the R1 or R2 transcripts, pAC-T730-FT (R1; virF +1 to +888) and pAC-T7-HH-21-FT (R2; virF +309 to +888) also harbored 3′ FLAG tags in *virF*. Upon IPTG induction, *virF* mRNA transcription from the T7 promoter was induced in *E. coli* BL21(D3) harboring either plasmid. PE analysis verified the expected 5′ ends of both transcripts (Fig. 7A).

VirF_21_ translation from the leaderless R2 mRNA was tested by immunoblot analysis on protein extracts after induction. VirF_21_ was detected in cells carrying pACT7-HH-21-FT, confirming that R2 is a leaderless translation-proficient mRNA (Fig. 7B, right panel). Translation of both VirF forms was observed in cells harboring pAC-T730-FT (Fig. 7B, left panel). *In vitro* translation in the PureSystem (30) was tested on R1 and R2 mRNAs carrying FLAG tag sequences. Translation products were analyzed with anti-FLAG antibodies. In agreement with the *in vivo* results, R1 mRNA supported translation of both VirF forms, whereas the leaderless R2 transcript only produced VirF_21_ (Fig. 7C). Furthermore, toeprint experiments on *in vitro*-transcribed *virF* R1 mRNA (start, +1) showed a strong RT stop near the 5′ end, indicating initiation complex formation at AUG_4_ (compare with Fig. 2C). In contrast, a specific toeprint was observed at position +326 for the llmRNA R2 (start, +309) (Fig. 7D). This toeprint was absent on R1 mRNA, indicating a strong preference for VirF_30_ translation from the full-length mRNA. Together, these results suggest that a new *virF* promoter generates a llmRNA variant (R2 mRNA) dedicated to the exclusive translation of VirF_21_.

## DISCUSSION

The complex regulatory cascade for activation of the *Shigella* virulence genes depends on the VirF protein (7). VirF is at the heart of the switch from the noninvasive to the invasive phenotype. Thus, it is not surprising that its expression is triggered by many environmental signals and that it is controlled at several levels (2, 4, 10, 17). Since its discovery, VirF was known to be present in three forms that differ in size: 30, 27, and 21 kDa (25). The smaller forms were ignored as likely degradation products. Here, we report that the VirF 21-kDa form is translated as an independent polypeptide. Our results address how the VirF_21_ variant is produced and suggest an autoregulatory role in *virF* expression*.* As a first step, we identified the translation start sites of VirF_30_ and VirF_21_. Of the three Met codons among the first four codons of the predicted *virF* ORF, only ATG4 was essential for VirF_30_ translation (Fig. 1 and 2). Replacement with GGG drastically reduced VirF, as monitored by Western blotting or β-galactosidase activity of *virF-lacZ* translational fusions (Fig. 2A and B). The identification of ATG4 as a start codon was further supported by toeprint analysis (Fig. 2C). The start codon consistent with the size of VirF_21_ is ATG81; accordingly, replacement with CTG blocks VirF_21_ production (Fig. 3B).

Interestingly, while the wt *virF* mRNA is translated into both VirF_30_ and VirF_21_ *in vivo*, a frameshift mutation upstream of ATG81 affects only the production of VirF_30_, and not that of VirF_21_. Thus, the two forms are independently translated (Fig. 3B); consequently, a derivative with either the FS mutation or the M81L substitution gives only VirF_21_ or only VirF_30_, respectively. β-Galactosidase fusion and immunoblot analyses (Fig. 3C) showed that the expression level of VirF_21_ under our experimental conditions is generally lower than that of VirF_30_.

VirF_21_ is clearly not functionally redundant with VirF_30_. Unlike VirF_30_, it does not restore the expression of VirF-regulated genes in a *virF*-defective *S. flexneri* mutant (Fig. 4). Instead, overexpression of VirF_21_ negatively autoregulates *virF* expression, reducing intracellular levels of VirF_30_ and causing reduced *virB* expression (Fig. 5B). This negative autoregulation is likely due to VirF_21_ binding to the *virF* promoter, as indicated by the position of a DNase I footprint (Fig. 5E) which is predicted to interfere with RNA polymerase access.

An arrangement based on a smaller protein controlling a larger one, with both of them encoded by the same gene, applies to Tn*5* transposase (31). The form of Tn*5* transposase lacking the first 55 amino acids posttranslationally forms nonproductive complexes with transposase, thus blocking its activity at IS*50* inverted repeats (31). Superficially similar in setup, the shorter VirF_21_ also lacks a large N-terminal portion of the longer VirF_30_ protein, but here, the shorter form alone is sufficient to exert control at the level of *virF* transcription (Fig. 5A). Though the known C-terminal DNA-binding domain is present in both VirF variants, our data suggest different DNA recognition preferences. Further work will test whether N-terminal sequences affect binding properties of VirF_30_ and whether protein folding differences in the shared domain can account for the observed specificity differences.

Since VirF_30_ and VirF_21_ originate from differential translation, we investigated the *virF* transcripts in more detail. Long (R1) and shorter (R2) *virF* mRNAs of lengths compatible with VirF_30_ and VirF_21_ were detected (Fig. 6A). Evaluation of deletions in the region upstream of the VirF_21_ ORF, along with PE analyses, identified a new gene-internal *virF* promoter that drives the transcription of the *virF* R2 mRNA. *In vivo* and *in vitro* data support that the leaderless R2 is translated into VirF_21_; plasmid vectors encoding R2 (start site, +309) support *in vivo* translation of VirF_21_ (Fig. 7B). Moreover, leaderless translation of VirF_21_ by R2 also occurs *in vitro* (Fig. 7C), and initiation complex formation occurs at the appropriate position (Fig. 7D).

In recent years, noncanonical translation initiation mechanisms have been reported, including so-called leaderless transcripts, i.e., those lacking a 5′-untranslated region (UTR) and an SD sequence (32–34). Most leaderless genes identified so far in *E. coli* reside in mobile DNA, including λ, P2, and Tn*1721*. The *virF* gene is also located within an IS-rich region on an extrachromosomal element, the large *Shigella*/EIEC invasive plasmid (9). Sequencing data for bacteria and archaea suggest that a leaderless model may not be uncommon (35, 36).

The mechanisms underlying synthesis and translation of llmRNAs are not yet fully understood. Vesper et al. (37) showed that induction of the MazEF toxin-antitoxin (TA) system in *E. coli* produces a leaderless mRNA population and, simultaneously, specialized “stress” ribosomes with a preference to translate proteins from llmRNAs. The endoribonuclease MazF cleaves single-stranded mRNAs, sometimes at ACA sequences upstream of AUG start codons, generating llmRNA. MazF also cleaves 16S rRNA, removing the anti-SD sequence required for translation on canonical mRNAs. Thereby, a subpopulation of ribosomes is generated for selective translation on llmRNA (37). It is well established that *Shigella* bacteria sense and respond to environmental conditions within and outside the host, with corresponding reprogramming of transcription. Since VirF_21_ modulates the intracellular level of VirF, this suggests that the transcription of the leaderless R2 mRNA could occur under conditions where the activation of the virulence program is undesirable. A possible coupling between stress conditions that might promote changes in R2 *virF* mRNA transcription and/or preferential translation of leaderless R2 mRNA and effects on virulence gene regulation is an exciting possibility that we intend to pursue. In particular, the environmental cues that may regulate transcription of the shorter *virF* mRNA, and the translation of VirF_21_ from the llmRNA under stress and infection-relevant conditions, will be addressed. In summary, this study has added new, entirely unexpected elements to the complex regulation of the *Shigella* virulence system and of its major regulator, the VirF protein.

## MATERIALS AND METHODS

### Oligodeoxyribonucleotides.

Oligodeoxynucleotides used in this study (see Table S1 in the supplemental material) were purchased from Metabion.

### Bacterial strains and general methods.

Strains used in this study are listed in Table S2 in the supplemental material. Cloning was performed wtih strain DH10b. *E. coli* strain P90CλB was obtained by transferring a P*_virB_*-*lacZ* fusion from plasmid pRS415 via homologous recombination to the *lac* transducing phage λRS45 and then integration (38) into the the λ *att* site of *E. coli* P90C. P90CλA was previously described (see Table S2). Strains M90T-F3xFT and M90T Fd(Δ*virF*) were previously constructed (21).

Bacteria were grown aerobically in LB medium at 37°C. Antibiotics and chemicals were used at the following concentrations: ampicillin, 50 µg/ml; chloramphenicol, 25 µg/ml; kanamycin, 30 µg/ml; streptomycin, 10 µg/ml; tetracycline, 5 µg/ml; 5-bromo-4-chloro-3-indolyl-β-d-galactopyranoside, 20 mg/ml. β-Galactosidase assays were performed as described elsewhere (39). Reported values represent the means of at least three separate measurements. DNA isolation, PCR, restriction digests, cloning, and other DNA manipulation methods were performed as described previously (39). Plasmids are listed in Table S3 in the supplemental material. In addition, plasmid constructions are detailed in Text S1 in the supplemental material.

### Analysis of *virF* mRNA.

*S. flexneri* M90T Fd (Δ*virF*) (Table S2) cells with or without pMYSH6504 were grown in LB broth at 37°C to an optical density at 600 nm of 0.4 to 0.5. Total RNA extraction and Northern blot assays with an α-^32^P-labeled *virF*-specific probe were performed as described elsewhere (21). Loading controls entailed rRNA staining. Radioactivity was quantified using a Typhoon 9200 instrument (GE Healthcare).

qRT-PCR was performed using Power SYBR green PCR master mix on a 7300 real-time PCR system (Applied Biosystems) as described previously (19). The levels of *virB*, *icsA*, and *lacZ* transcripts were analyzed using the 2−^ΔΔ*CT*^ (cycle threshhold [*C_T_*]) method (40), and results are reported that the fold increase relative to the reference. Primers for *mdh* (endogenous control) and for *virB*, *icsA*, and *lacZ* transcripts were designed by using Primer Express software v2.0 and validated. The following oligonucleotides were used (see Table S1 in the supplemental material): *mdhQF/mdhQR*, *virBQF/virBQR*, *icsAQL/icsAQR*, and *lacZQF/lacZQR*.

### Primer extensions.

Total RNA from exponentially growing plasmid-carrying *E. coli* strains was extracted (41). Total RNA (10 to 20 µg) was hybridized with 5′-^32^P-labeled ML-512 and ML-1314 primers. Reverse transcription experiments were done at 42°C using the reverse transcriptase ImProm-II (Promega). Reaction products were analyzed on an 8% polyacrylamide gel in parallel with sequencing reaction products obtained using the same primers.

### DNase I footprinting.

Supercoiled plasmid pMYSH6504 (42) (200 ng/sample) was preincubated for 20 min at room temperature with the indicated volumes of the translation mixture, which contained VirF_21_ or control (no-template) PureSystem reagent in 30 µl of binding buffer (40 mM Tris-HCl [pH 8.0], 50 mM KCl, 10 mM Mg-acetate, and 0.5 mM dithiothreitol). The DNA-protein complex was incubated with 1 U of DNase I for 40 s. After stopping the reaction, the DNA was precipitated and separately analyzed by primer extension on either DNA strand with 3 pmol of 5′-end-labeled primers ML-U30 or ML-U29 as described previously (14). The extension products and corresponding sequencing reactions were run on 7% sequencing gels and then fixed for 5 min (10% ethanol–6% acetic acid) and dried. Signals were detected using a phosphorimager screen.

### Immunodetection of VirF protein.

Western blot assays were carried out as described in reference 21. Incubation with primary antibodies (polyclonal halon anti-VirF, anti-FLAG [Sigma F1804]) was at 4°C in phosphate-buffered saline–Tween (PBS-T) containing 2% dried skim milk. Membranes were washed and incubated at room temperature for 1 h with a secondary anti-rabbit (1:10,000) or anti-mouse (1:5,000) horseradish peroxidase-conjugated antibody in PBS-T. After washing with PBS-T, membranes were developed for 5 min for enhanced chemiluminescence and visualized on a ChemiDoc XRS+ system.

### RNA *in vitro* transcription.

The *virF*-3XFT mRNAs R1 and R2 were transcribed for *in vitro* translation and toeprint assays. For *virF* mRNA R1-3XFT (start, +1), DNA templates contained a T7 promoter (PCR with primers ML-U1/ML-982). For *virF* mRNA R2-3XFT (start, +309), a fragment with a T7 promoter and a hammerhead ribozyme sequence in front of the *virF* sequence was produced by PCR (primers ML-U20/ML-982) on pAC-T7-HH-21-FT as the template (for the hammerhead sequence, Fig. S5 in the supplemental material). DNA templates were *in vitro* transcribed as described in reference 43. To obtain *virF* R2-3XFT, an additional ribozyme self-cleavage step was performed after *in vitro* transcription according methods described previously (44).

### Toeprint assay.

Toeprint assays were performed as in reference 45. Aliquots of 0.2 pmol of unlabeled *virF*-3xft mRNAs R1 and R2 were annealed with 0.5 pmol 5′-end-labeled ML-U25 or ML-U26 primer in water at 95°C for 1 min and chilled on ice for 2 min. After addition of renaturing buffer (20 mM Tris-HCl [pH 7.5], 20 mM MgCl_2_, 100 mM NH_4_Cl) and incubation for 10 min at 37°C, 2 pmol of 30S ribosomal subunits was added. After 15 min, 4 pmol of tRNA-fMet was added, and incubation continued for 20 min before cDNA synthesis with avian myeloblastosis virus reverse transcriptase (7.5 U; Invitrogen) and deoxynucleoside triphosphates (100 nM). Reactions were stopped by phenol-chloroform-isoamyl alcohol extraction followed by ethanol precipitation. The cDNAs and sequencing reactions were run on 8% denaturing polyacrylamide gels that were fixed for 5 min (10% ethanol–6% acetic acid) and dried for 1 h at 80°C. Signals were detected using a phoshorimager screen.

### *In vitro* translation.

To generate VirF_21_ for DNase I footprinting, 500 ng of a PCR product containing a T7 promoter and the *virF_21_* coding sequence was used as the template in the PureSystem Express (New England BioLabs [NEB]) transcription-translation system at 37°C for 4 h. VirF_21_ translation was analyzed by immunoblotting using anti-VirF antibodies (see Fig. S4 in the supplemental material). For the *in vitro* translation of different *virF* mRNAs (Fig. 7C), each purified transcript was denatured for 2 min at 95°C, chilled for 1 min on ice, diluted in TMN (20 mM Tris-HCl [pH 7.5], 10 mM MgCl_2_, 150 mM NaCl), and incubated for 15 min at room temperature. *In vitro* translation (mRNA at 50 nM) was performed with the PureSystem Express (NEB) translation system at 37°C. Translation products were analyzed by immunoblotting with anti-FLAG antibodies.

## SUPPLEMENTAL MATERIAL

Figure S1  *In vivo* levels of *virB* expression as a function of VirF_30_ were monitored by qRT-PCR in a *virF*-defective *S. flexneri* strain (M90TFd) transformed with pMYSH6504, pF-M81L, or pF-M81I. Expression levels in M90TFd were used as the control. At least three wells were run for each sample, and error bars display the calculated maximum (RQMax) and minimum (RQMin) levels of the standard error of the mean expression level (RQ value). Download Figure S1, PDF file, 0.1 MB

Figure S2  Western blot analysis (with anti-VirF halon serum) of whole extracts of DH10b carrying pMYSH6504 (expressing both VirF_30_ and VirF_21_), pF-M81L (expressing only VirF_30_), or pF-FS (expressing only VirF_21_). The asterisks indicate unspecific cross-hybridization with other proteins in the extracts. Download Figure S2, PDF file, 0.1 MB

Figure S3  *In vivo* levels of *groEL* and *htpG* mRNAs as a function of VirF_30_ or VirF_21_ were monitored by real-time PCR in a *virF*-defective *S. flexneri* strain (M90TFd) transformed with pMYSH6504, pF-M81L, or pF-FS. Expression levels monitored in M90T were used as a control. At least three wells were run for each sample, and error bars display the calculated maximum (RQMax) and minimum (RQMin) levels, which represent standard errors of the mean expression levels (RQ values). The following oligonucleotides were used (see Table S1): *groELQL*/*groELQR* and *htpGQL*/*htpGQR*. Download Figure S3, PDF file, 0.1 MB

Figure S4  *In vitro* translation of VirF_21_ protein used for the DNase I footprinting experiment (Fig. 5E). *In vitro* transcription-translation was done in the PureExpress system using a PCR-generated DNA template with a T7 promoter immediately followed by the *virF_21_* ORF sequence. A 2.5-µl aliquot of the reaction was loaded and the protein was detected with a polyclonal anti-VirF halon antibody. In parallel, 2.5 µl of extract minus template was analyzed. Asterisk, unspecific cross-hybridization with proteins in the reaction mixture. Download Figure S4, PDF file, 0.1 MB

Figure S5  Secondary structure of the hammerhead ribozyme used to generate the *virF* R2 leaderless mRNA (start, +309). The ribozyme sequence was designed according to methods described elsewhere (J. M. Avis, G. L. Conn, S. C. Walker SC, Methods Mol Biol **941:**83–98, 2012, http://dx.doi.org/10.1007/978-1-62703-113-4_7). Download Figure S5, PDF file, 0.1 MB

Table S1  Oligodeoxyribonucleotides used in this study. Table S1, PDF file, 0.04 MB

Table S2  Strains used in this study. Table S2, PDF file, 0.1 MB

Table S3  Plasmids used in this study. Table S3, PDF file, 0.05 MB

Text S1  Plasmid constructions and related references. Download Text S1, PDF file, 0.02 MB
