# Supplementary figures and images for: One Gene and Two Proteins: a Leaderless mRNA Supports the Translation of a Shorter Form of the Shigella VirF Regulator
Source: mBio. 2016 Nov 8;7(6):e01860-16. doi: 10.1128/mBio.01860-16 (PMC5101355; doi:10.1128/mBio.01860-16)

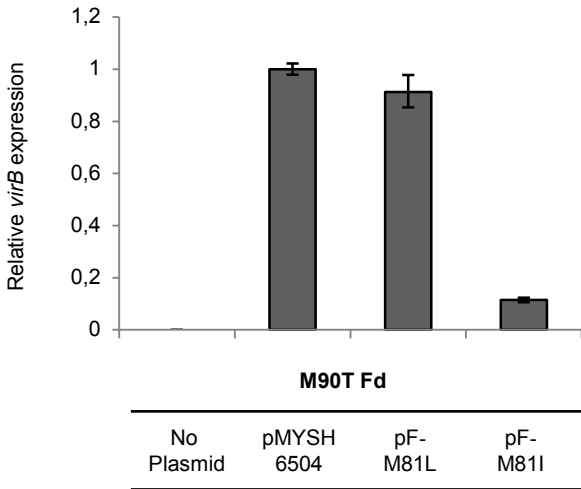

Supplement: Figure S1 — In vivo levels of virB expression as a function of VirF30 were monitored by qRT-PCR in a virF-defective S. flexneri strain (M90TFd) transformed with pMYSH6504, pF-M81L, or pF-M81I. Expression levels in M90TFd were used as the control. At least three wells were run for each sample, and error bars display the calculated maximum (RQMax) and minimum (RQMin) levels of the standard error of the mean expression level (RQ value). Download [file mbo006163057sf1.pdf]

No plasmid

pMYSH6504

pF-M81L

pF-FS

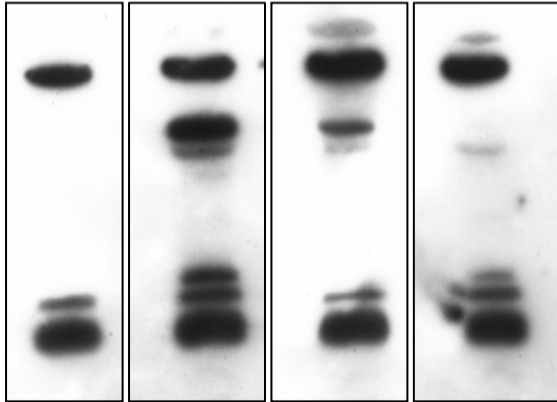

\*

VirF<sub>30</sub>

VirF<sub>21</sub>

\*

\*

Supplement: Figure S2 — Western blot analysis (with anti-VirF halon serum) of whole extracts of DH10b carrying pMYSH6504 (expressing both VirF30 and VirF21), pF-M81L (expressing only VirF30), or pF-FS (expressing only VirF21). The asterisks indicate unspecific cross-hybridization with other proteins in the extracts. Download [file mbo006163057sf2.pdf]

$p < 0,01$

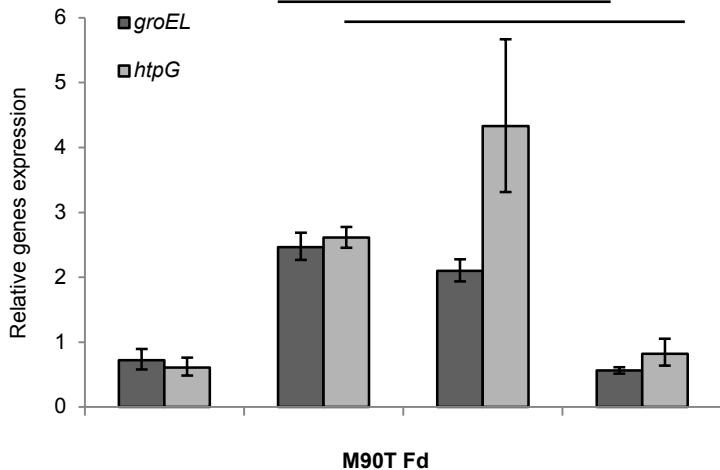

|                    | M90T Fd    |            |         |       |
|--------------------|------------|------------|---------|-------|
|                    | No Plasmid | pMYSH 6504 | pF-M81L | pF-FS |
| VirF <sub>30</sub> | -          | +          | +       | -     |
| VirF <sub>21</sub> | -          | +          | -       | +     |

Supplement: Figure S3 — In vivo levels of groEL and htpG mRNAs as a function of VirF30 or VirF21 were monitored by real-time PCR in a virF-defective S. flexneri strain (M90TFd) transformed with pMYSH6504, pF-M81L, or pF-FS. Expression levels monitored in M90T were used as a control. At least three wells were run for each sample, and error bars display the calculated maximum (RQMax) and minimum (RQMin) levels, which represent standard errors of the mean expression levels (RQ values). The following oligonucleotides were used (see Table S1): groELQL/groELQR and htpGQL/htpGQR. Download [file mbo006163057sf3.pdf]

-

+

T7 - virF<sub>21</sub> PCR

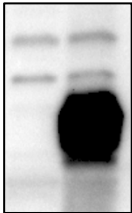

\*

\*

VirF<sub>21</sub>

Supplement: Figure S4 — In vitro translation of VirF21 protein used for the DNase I footprinting experiment (Fig. 5E). In vitro transcription-translation was done in the PureExpress system using a PCR-generated DNA template with a T7 promoter immediately followed by the virF21 ORF sequence. A 2.5-µl aliquot of the reaction was loaded and the protein was detected with a polyclonal anti-VirF halon antibody. In parallel, 2.5 µl of extract minus template was analyzed. Asterisk, unspecific cross-hybridization with proteins in the reaction mixture. Download [file mbo006163057sf4.pdf]

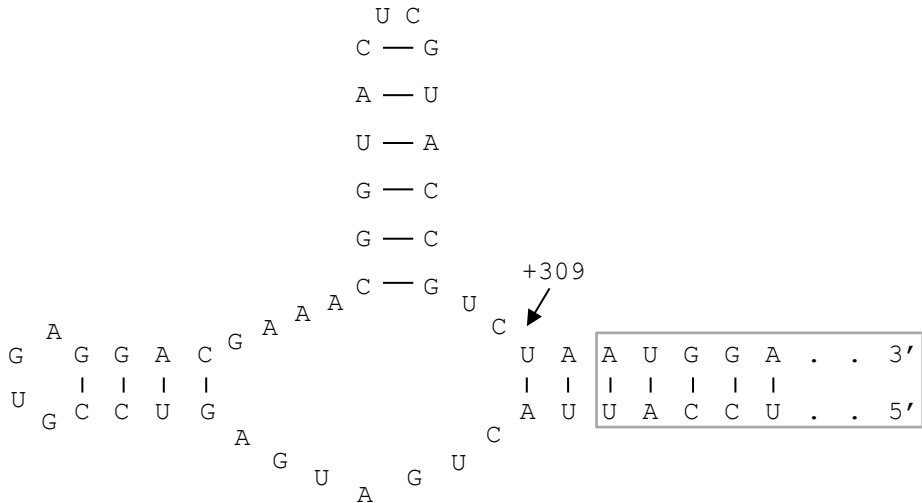

Supplement: Figure S5 — Secondary structure of the hammerhead ribozyme used to generate the virF R2 leaderless mRNA (start, +309). The ribozyme sequence was designed according to methods described elsewhere (J. M. Avis, G. L. Conn, S. C. Walker SC, Methods Mol Biol 941:83–98, 2012, http://dx.doi.org/10.1007/978-1-62703-113-4_7). Download [file mbo006163057sf5.pdf]
