# Supplementary material for: One Gene and Two Proteins: a Leaderless mRNA Supports the Translation of a Shorter Form of the Shigella VirF Regulator
Source: mBio. 2016 Nov 8;7(6):e01860-16. doi: 10.1128/mBio.01860-16 (PMC5101355; doi:10.1128/mBio.01860-16)
Supplement: Table S1 — Oligodeoxyribonucleotides used in this study. [file mbo006163057st1.pdf]

Table S1. Oligodeoxyribonucleotides used in this study

Name	Sequence (5'- 3')	Used for
ML-296	ATATAGTTTGGTTATTCTGTTGAATTTATGATGGATGG GGGACATAAAAACAAAATAGAT	SDM[a]
ML-297	ATCTATTTTGTTTTTATGTCCCCCATCCATCATAAATTC AACAGAATAACCAAACTATAT	SDM
ML-394	CCTTGACAGAAATTTATTATTAAGCATTATTAGAATAC TGGAACCAATTTATTCATTTCA	SDM
ML-395	TGAAATGAATAAATTGGTTCCAGTATTCTAATAATGCT TAATAATAAATTTCTGTCAAGG	SDM
ML-397	CCTTGACAGAAATTTATTATTAAGCATTATTAGAATAA TCGAACCAATTTATTCATTTCA	SDM
ML-398	TGAAATGAATAAATTGGTTCGATTATTCTAATAATGCT TAATAATAAATTTCTGTCAAGG	SDM
ML-331	AAGGGCAAATTGCTTTTATAGAGGCGAAATATACAAA TAAACGTCTC	SDM
ML-332	GAGACGTTTATTTGTATATTTCGCCTCTATAAAAGCAA TTTGCCCTT	SDM[a]
ML-1344	TGAATAAATTGGTTCCATTATTCTAATAACGCTTAATAATAA ATTTCTGTCAAGGCTTA	SDM[a]
ML-1345	TAAGCCTTGACAGAAATTTATTATTAAGCGTTATTAGAATAA TGGAACCAATTTATTCA	SDM
virBQF	CCAAGTTCTCGGATGCTATGC	qRT-PCR
virBQR	CTCTTGATGCCAGAAAACTAGCAA	qRT-PCR
icsAQF	TGATGGACTTTCTCCCTTGGG	qRT-PCR
icsAQR	TACCACGCATCCATTCCATCT	qRT-PCR
groELQF	TCTGTGGCTGGCCTGATGA	qRT-PCR
groELQR	TTTTTCGGCAGGTCGGTAAC	qRT-PCR
htpGQF	CAACCGCGTTGAAGGTAAGC	qRT-PCR
htpGQR	GACGGGATGTACAGCAGGCT	qRT-PCR
lacZQF	CCCATCTACACCAACGTGACC	qRT-PCR
lacZQR	AACAAACGGCGGATTGACC	qRT-PCR
mdhQF	CCTGGTACAGCAAGTTGCG	qRT-PCR
mdhQR	CGTAGGCACATTCGACAACG	qRT-PCR
ML-512	TCAACAATCTTCCTTATCTGATC	Primer Extension
ML-1314	TTAACCCCCTTTTCTCCTC	Primer Extension
ML-U25	GTTTCATTGCCTGAGCTA	Toeprinting
ML-U26	CAAATCGATAGAAACCTC	Toeprinting
GN20	TACGGATCCATTCTTAATAACGGAAAG	Northern
FsinR	GGGATCCCCATCTGGC	Northern
ML-333	NNNGGATCCATGGGACATAAAAACAAAATAG	*virF*$_{30}$ pAC-30 cloning
ML-335	NNNGGATCCATGGAACCAATTTATTCATTTC	*virF*$_{21}$ pAC-21 cloning
ML-336	NNNGGATCCGGAAAACCCATCTGGCAA	*virF*$_{30}$ pAC30 and *virF*$_{21}$ pAC21 cloning
ML-438	NNNGGATCCTTAATACGACTCACTATAGACAGAAATT TCTTAGTTACTCT	T7-*virF*$_{30}$ pAC-30-FT cloning
ML-566	TTACTATTTATCGTCGTCATCTTTGTAGTCAAATTTTTT ATGATATAAGTAAAATTTCA	T7-*virF*$_{30}$ pAC-30-FT cloning
ML-U6[b]	TCGACTTAATACGACTCACTATAGGGTCCATTACTGATGAGT CCGTGAGGACGAAACGGTACTCGGTACCGTCG	T7-HH pAC-T7-HH cloning
ML-U13[b]	GATCCGACGGTACCGAGTACCGTTTCGTCCTCACGGACTCAT CAGTAATGGACCCTATAGTGAGTCGTATTAAG	T7-HH pAC-T7-HH cloning
ML-U7	NNNGGTACCGTCTAATGGAACCAATTTATTCATTTC	*virF*+309-FT pAC-T7-HH-21-FT cloning

ML-U8	NNNGGATCCCAGTCATAGCCGAATAGCCT	*virF*+309-FT pAC-T7-HH-21-FT cloning
FustradFL	TTAGGATCCATATCCATCATAAATTC	*virF* pFL-4A fusion cloning
FustradFS	ATAGGATCCATAAATTCAACAGAATA	*virF* pFL-1A fusion cloning
ML-496	CTGGATCCTCCGCTTCCTTTAGCAGC	*virF-lacZ* fusion cloning
ML-330	NNNGGATCCATAGAAACCTCCTCCTCAGA	*virF-lacZ* fusion cloning
ML-334	NNNGGATCCATGACGGTTAGCTCAGGCAATG	Transcriptional *virF-lacZ* fusion cloning
ML-1342	NNNGGATCCGAATAATGGAACCAATTTATTC	Transcriptional *virF-lacZ* fusion cloning
ML-1343	NNNGATCCGAGCGAAATATACAAATAAACG	Transcriptional *virF-lacZ* fusion cloning
bx5	GGAGCTCTCACATCAG	*virB-lacZ* p_{virB}-lacZ fusion cloning
bx6	GTCGTTGCACAAATCC	*virB-lacZ* p_{virB}-lacZ fusion cloning
ML-U1	GAAATTAATACGACTCACTATAGACAGAAATTTCTTAGTTACT	*virF* R1 mRNA template for *in vitro* transcription
ML-U20	TAATACGACTCACTATAGGG	*virF* R2 mRNA template for *in vitro* transcription
ML-982	NNNGGATCCTGAGGGGATCTTGAAGTTCC	*virF* R1 and R2 mRNA template for *in vitro* transcription
QH7-	TAGGGATCCAAGCGAACCTTTATATC	*virF-lacZ* pvirF-lacZ cloning
ML-U3	GAAATTAATACGACTCACTATAGGGAGATTATAAGCCTTGACAGA	$VirF_{21}$ PCR template for *in vitro* translation
ML-U28	GGGATCCCCATCTGGC	$VirF_{21}$ PCR template for *in vitro* translation
ML-U30	GGTTATAGTCCCTTTCAGTGCA	DNase I footprinting
ML-U29	CAAGCGAACCTTTATATC	DNase I footprinting

[a]SDM: Site Directed Mutagenesis

[b]The underlined nucleotides specify the hammerhead sequence used for mRNA cleavage
