# Supplementary material for: One Gene and Two Proteins: a Leaderless mRNA Supports the Translation of a Shorter Form of the Shigella VirF Regulator
Source: mBio. 2016 Nov 8;7(6):e01860-16. doi: 10.1128/mBio.01860-16 (PMC5101355; doi:10.1128/mBio.01860-16)
Supplement: Table S2 — Strains used in this study. [file mbo006163057st2.pdf]

**Table S2.** Strains used in this study

| Strain | Description | Source/Reference |
|---|---|---|
| M90T | *S. flexneri 5a* | (1) |
| M90T Fd | M90T *virF* | (2) |
| M90T F-3xFT | M90T *virF*-3xFT | (2) |
| DH10b | *E. coli K12* | (3) |
| P90C | *E. coli* | (4) |
| P90C λB | P90C derivative strain harboring a chromosomally located copy of a *virB-lacZ* fusion | This study |
| P90CλA | P90C derivative strain harboring a chromosomally located copy of an *icsA-lacZ* fusion; renamed from ULS1127 | (5) |
| BL21 (DE3) | *E. coli* strain for protein expression | (6) |

REFERENCES


1. **Sansonetti PJ**, **d'Hauteville H**, **Formal SB**, **Toucas M**. Plasmid-mediated invasiveness of "*Shigella*-like" *Escherichia coli*. Ann Microbiol (Paris) **133**:351–5.

2. **Leuzzi A**, **Di Martino ML**, **Campilongo R**, **Falconi M**, **Barbagallo M**, **Marcocci L**, **Pietrangeli P**, **Casalino M**, **Grossi M**, **Micheli G**, **Colonna B**, **Prosseda G**. 2015. Multifactor Regulation of the MdtJI Polyamine Transporter in Shigella. PLoS One **10**:e0136744.

3. **Durfee T**, **Nelson R**, **Baldwin S**, **Plunkett G**, **Burland V**, **Mau B**, **Petrosino JF**, **Qin X**, **Muzny DM**, **Ayele M**, **Gibbs RA**, **Csörgo B**, **Pósfai G**, **Weinstock GM**, **Blattner FR**. 2008. The complete genome sequence of *Escherichia coli* DH10B: insights into the biology of a laboratory workhorse. J Bacteriol **190**:2597–606.

4. **Sasakawa C**, **Komatsu K**, **Tobe T**, **Suzuki T**, **Yoshikawa M**. 1993. Eight genes in region 5 that form an operon are essential for invasion of epithelial cells by *Shigella flexneri* 2a. J Bacteriol **175**:2334–46.

5. **Giangrossi M**, **Prosseda G**, **Tran CN**, **Brandi A**, **Colonna B**, **Falconi M**. 2010. A novel antisense RNA regulates at transcriptional level the virulence gene *icsA* of *Shigella flexneri*. Nucleic Acids Res **38**:3362–75.

6. **Jeong H**, **Barbe V**, **Lee CH**, **Vallenet D**, **Yu DS**, **Choi S-H**, **Couloux A**, **Lee S-W**, **Yoon SH**, **Cattolico L**, **Hur C-G**, **Park H-S**, **Ségurens B**, **Kim SC**, **Oh TK**, **Lenski RE**, **Studier FW**, **Daegelen P**, **Kim JF**. 2009. Genome sequences of *Escherichia coli* B strains REL606 and BL21(DE3). J Mol Biol **394**:644–52.
