# Supplementary material for: One Gene and Two Proteins: a Leaderless mRNA Supports the Translation of a Shorter Form of the Shigella VirF Regulator
Source: mBio. 2016 Nov 8;7(6):e01860-16. doi: 10.1128/mBio.01860-16 (PMC5101355; doi:10.1128/mBio.01860-16)
Supplement: Table S3 — Plasmids used in this study. [file mbo006163057st3.pdf]

Table S3. Plasmids used in this study

Plasmid name	Description	Source/ Reference
pMYSH6504	pBR322 derivative carrying *S. flexneri virF* gene	(1)
pF-M4G	pMYSH6504 derivative with ATG to GGG mutation at positions 71-73 in *virF* (primer pairs used: ML-296 /ML-297)	This study
pF-M81L	pMYSH6504 derivative with ATG to CTG mutation at positions 311-313 in *virF* (ML-394/ML-395)	This study
pF-M81I	pMYSH6504 derivative with ATG to ATC mutation at positions 311-313 in *virF* (ML-397/ML-398)	This study
pF-FS	pMYSH6504 derivative with insertion G after position 208 in *virF* (ML-331/ML-332)	This study
p_{virB}-lacZ	*virB-lacZ* transcriptional fusion in pRS415 (bx5/bx6)	This study
pFL-1A	*virF-lacZ* translational fusion, *virF* (-289 to +64), from Met1 in frame with the CDS of *lacZ* (ML-496/ML- FustradFS)	This study
pFL-4A	*virF-lacZ* translational fusion, *virF* (-289 to +73) from Met4 in frame with the CDS of *lacZ* (ML-496/ML- FustradFL)	This study
pFL-M4G	pFL-4A with mutations ATG to GGG at positions 71-73) in *virF* (ML- ML-296 /ML-297)	This study
pVirF-lacZ	*virF(wt)-lacZ* translational fusion in pRS414 (pos: -289 to +104) (ML-496/Qh7)	This study
pRS-6504	*virF(wt)-lacZ* translational fusion in pRS414 (pos: -289 to +405) (ML-496/ML-330)	This study
pRS-M81L	*virF*(M81L)-*lacZ* translational fusion in pRS414 (ML-496/ML-330)	This study
pRS-M4G	*virF*(M4G)-*lacZ* translational fusion in pRS414 (ML-496/ML-330)	This study
pRS-FS	*virF*(FS)-*lacZ* translational fusion in pRS414 (ML-496/ML-330)	This study
pAC-30	pGIP7 derivative carrying $virF_{30}$M81L ORF (ML-333/ML-336)	This study
pAC-21	pGIP7 derivative carrying $virF_{21}$ ORF (ML-335/ML-336)	This study
pRS-F(+70)	*virF-lacZ* transcriptional fusion in pRS415 (pos: +70 to +405) (ML-333/ML-330)	This study
pRS-F(+145)	As pRS-F(+70) but from +145 to +405 (ML-334/ML-330)	This study
pRS-F(+205)	As pRS-F(+70) but from +205 to +405) (ML-1343/ML-330)	This study
pRS-F(+305)	As pRS-F(+70) but from +305 to +405) (ML-1342/ML-330)	This study
pRS-F(+205 -10mut)	pRS-F(+205) derivative with mutations CATTAT to CGTTAT at position +298 to +303 in *virF* (ML-1344/ML-1345)	This study
pAC-T730-FT	pACYC184 derivative carrying *virF* sequence (+1) under the control of a T7 promoter (ML-438/ML-566)	This study
pAC-T7-HH	pACYC184 derivative with a T7 promoter and a specific hammerhead ribozyme for *virF* (ML-U6/ML-U13)	This study
pAC-T7-HH-FT	pACYC184 derivative carrying *virF* sequence (+309) preceded by a T7 promoter and a specific hammerhead ribozyme sequence (ML-U7/ML-U8)	This study
pGIP7	pACYC184 derivative carrying *lacI* gene and P_{lac}	(2)
pACYC184	Medium copy number cloning vector	(3)
pRS415	LacZ transcriptional fusion vector	(4)
pRS414	LacZ translational fusion vector	(4)

REFERENCES

1. **Sakai T**, **Sasakawa C**, **Makino S**, **Kamata K**, **Yoshikawa M**. 1986. Molecular cloning of a genetic determinant for Congo red binding ability which is essential for the virulence of *Shigella flexneri*. Infect Immun **51**:476–82.

2. **Falconi M**, **Prosseda G**, **Giangrossi M**, **Beghetto E**, **Colonna B**. 2001. Involvement of FIS in the H-NS-mediated regulation of *virF* gene of *Shigella* and enteroinvasive *Escherichia coli*. Mol Microbiol **42**:439–52.

3. **Chang AC**, **Cohen SN**. 1978. Construction and characterization of amplifiable multicopy DNA cloning vehicles derived from the P15A cryptic miniplasmid. J Bacteriol **134**:1141–56.

4. **Simons RW**, **Houman F**, **Kleckner N**. 1987. Improved single and multicopy lac-based cloning vectors for protein and operon fusions. Gene **53**:85–96.
