# Supplementary material for: One Gene and Two Proteins: a Leaderless mRNA Supports the Translation of a Shorter Form of the Shigella VirF Regulator
Source: mBio. 2016 Nov 8;7(6):e01860-16. doi: 10.1128/mBio.01860-16 (PMC5101355; doi:10.1128/mBio.01860-16)
Supplement: Text S1 — Plasmid constructions and related references. Download [file mbo006163057s1.pdf]

Plasmid constructions

Plasmids pF-M4G, pF-M84L, pF-M84I, and pF-FS, are derivatives of pMYSH6504, a vector carrying the entire *virF* gene of *S. flexneri* (1). Mutations in *virF* were introduced with the Quick-change II XL mutagenesis kit (Agilent) as detailed in Table S3. Plasmids pFL-1A and pFL-4A are *virF-lacZ* in-frame fusions after the first (-289 to +64) or the fourth (-289 to +73) ATG codon, respectively; pFL-M4G is a pFL-4A derivative with the M4G substitution (primers: ML-296/ML-297); Translational *virF–lacZ* fusions were constructed as follows: p_{virF}-lacZ was constructed by inserting a fragment from -289 to +104 of *virF* (relative to +1 of the transcript) in frame to *lacZ* in the vector pRS414 (2) (primers: ML-496/ML-QH7). To obtain pRS-6504, pRS-M81L, pRS-M4G and pRS-FS, fragments from -289 to +405 of *virF* were PCR-amplified on pMYSH6504, pF-M81L, pF-M4G, pF-FS, and pF-M81L-FS template DNA, respectively, using oligo pairs reported in Table S3, and inserted in-frame to *lacZ* gene in pRS414.

Plasmid p_{virB}-lacZ carries a *virB-lacZ* translational fusion and was obtained by cloning a fragment containing the *virB* promoter region and the first 7 codons of *virB* (primers: bx5-bx6) into pRS414.

Transcriptional *virF-lacZ* fusions: *virF* fragments from +70 to + 405, +145 to +405, + 205 to + 405, and +305 to + 405, were PCR-amplified on pMYSH6504 and cloned upstream of the *lacZ* gene in pRS415 (2), resulting in pRS-F(+70), pRS-F(+145), pRS-F(+205), pRS-F(+305), respectively (primers: Table S3). Mutation of the –10 element (CATTAT to CGTTAT) in the plasmid pRS-F(+205 –10mut) was introduced with the Quick-change II XL mutagenesis kit (Agilent) as detailed in Table S3.

Plasmids pAC-30 and pAC-21 are pGIP7 (3) derivatives encoding $VirF_{30}$ (primers : ML-333/ML-336) or $VirF_{21}$ (primers: ML-335/ML-336), respectively, under the control of a Ptac promoter; pAC-T730-FT and pAC-T7-HH-21-FT are pACYC184-derivatives with T7 promoters (primers: ML-438/ML-566): the former carries the full *virF* sequence (start +1), the latter has the shorter *virF* sequence (start +309), preceded by a hammerhead ribozyme sequence to produce the leaderless mRNA R2. Briefly, complementary oligos containing the T7 promoter and hammerhead sequence

were annealed and cloned in pACYC184 (primers : ML-U6/ML-U13). The hammerhead sequence was designed as in (4) and is shown in Figure S4. The resulting plasmid was digested with *Kpn*I/*Bam*HI taking advantage of the *Kpn*I site present in the hammerhead sequence, and a PCR product of *virF* (starting at +309) (primers: ML-U7/ML-U8) followed by the 3xFT was inserted, generating pAC-T7-HH-21-FT.

REFERENCES

1. **Sakai T**, **Sasakawa C**, **Makino S**, **Kamata K**, **Yoshikawa M**. 1986. Molecular cloning of a genetic determinant for Congo red binding ability which is essential for the virulence of *Shigella flexneri*. Infect Immun **51**:476–82.
2. **Simons RW**, **Houman F**, **Kleckner N**. 1987. Improved single and multicopy lac-based cloning vectors for protein and operon fusions. Gene **53**:85–96.
3. **Falconi M**, **Prosseda G**, **Giangrossi M**, **Beghetto E**, **Colonna B**. 2001. Involvement of FIS in the H-NS-mediated regulation of *virF* gene of *Shigella* and enteroinvasive *Escherichia coli*. Mol Microbiol **42**:439–52.
4. **Avis JM**, **Conn GL**, **Walker SC**. 2012. Recombinant and In Vitro RNA Synthesis. Humana Press, Totowa, NJ.
